# Facilitation of animals is stronger during summer marine heatwaves and around morphologically complex foundation species

**DOI:** 10.1002/ece3.10512

**Published:** 2023-09-17

**Authors:** Shinae Montie, Mads S. Thomsen

**Affiliations:** ^1^ Marine Ecology Research Group, School of Biological Sciences University of Canterbury Christchurch New Zealand; ^2^ Aarhus University Department of Ecoscience Roskilde Denmark

**Keywords:** active heating experiment, biodiversity, epifauna, facilitation cascade, foundation species, habitat cascade, heatwaves, invertebrates, seaweed mimics, subtidal habitat

## Abstract

Foundation species create biogenic habitats, modify environmental conditions, augment biodiversity, and control animal community structures. In recent decades, marine heatwaves (MHWs) have affected the ecology of foundation species worldwide, and perhaps also their associated animal communities. However, no realistic field experiment has tested how MHWs affect animals that live in and around these foundation species. We therefore tested, in a four‐factorial field experiment, if colonisation by small mobile marine animals (epifauna) onto plates with attached single versus co‐occurring foundation species of different morphological complexities, were affected by 3–5°C heating (that mirrored a recent extreme MHW in the study area) and if the heating effect on the epifauna varied within and between seasons. For this experiment mimics of turf seaweed represented the single foundation species and holdfasts of seven common canopy‐forming seaweed represented the co‐occurring foundation species with different morphological complexities. We found that the taxonomic richness and total abundance of epifauna, dominated by copepods, generally were higher on heated plates with complex seaweed holdfasts in warmer summer trials. Furthermore, several interactions between test‐factors were significant, e.g., epifaunal abundances, were, across taxonomic groups, generally higher in warmer than colder summer trials. These results suggest that, in temperate ecosystems, small, mobile, short‐lived, and fast‐growing marine epifauna can be facilitated by warmer oceans and morphologically complex foundation species, implying that future MHWs may increase secondary production and trophic transfers between primary producers and fish. Future studies should test whether these results can be scaled to other ecological species‐interactions, across latitudes and biogeographical regions, and if similar results are found after longer MHWs or within live foundation species under real MHW conditions.

## Graphical abstract

Foundation species facilitate other organisms, but stressors like heatwaves affect their ecology. How facilitative processes are impacted during heatwaves is unknown. Here, heatwaves were experimentally induced with co‐occurring spatiotemporal conditions. We found facilitation was higher during heatwaves, warmer summer periods, and around complex habitat. These conditions may increase secondary production rates in the temperate marine environment.

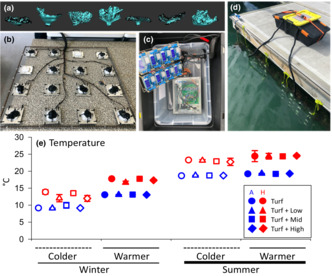

## 1 INTRODUCTION

Primary foundation species, such as trees, corals, and seaweed, can increase ecological performance (like colonisation, survival, abundances, reproduction, etc.) and thereby facilitate animals, by increasing the structural space to live in and around. Foundation species also reduce environmental stress and consumer pressures compared to habitats that lack foundation species (Bruno et al., 2003; Ellison et al., 2005; Jones et al., 1994). Furthermore, primary foundation species can provide living space to secondary foundation species such as epiphytes attached to aquatic plants and trees, mussels embedded among marsh grasses, or canopy‐forming seaweed overgrowing turf‐forming species (Altieri et al., 2007; Angelini et al., 2011; Gribben et al., 2019). When primary and secondary foundation species co‐occur, the amount and complexity of biogenically formed three‐dimensional living spaces often increase (compared to when the primary foundation species exist in isolation) and thereby augment the biodiversity of habitat‐associated animals (Navarro‐Barranco et al., 2022; Thomsen et al., 2022; Tokeshi & Arakaki, 2012). Today, facilitation of animals from co‐occurring foundation species has been documented across habitats, ecosystems, and bioregions (Angelini et al., 2011; Kazanidis et al., 2022; Thomsen et al. 2018b). It has also been shown that facilitation of animals can vary with the density of the foundation species, the elevation and latitudes where the foundation species live (Angelini et al., 2015; Bishop et al., 2012; Ravaglioli et al., 2021), and the morphological complexity of the co‐occurring foundation species (Thomsen et al., 2022). However, little is known about the temporal dynamics of facilitation of animals associated with co‐occurring foundation species, e.g., if facilitation changes within and between seasons (Gribben et al., 2019; Smith et al., 2023) or during periods of unusually high temperature (Chen et al. 2021b). The latter is particularly important to understand because burning of fossil fuels and associated atmospheric greenhouse gases are increasing ocean temperatures (Smith et al., 2023). Furthermore, recent studies have shown that, superimposed on global warming, marine heatwaves (MHWs), i.e. discrete periods of unusually high sea surface temperature (Hobday et al., 2016), have become more frequent and intense (Frölicher et al., 2018; Oliver et al., 2019, 2021; Thoral et al., 2022), sometimes with severe impacts on seaweed foundation species, like kelp and large fucoids (Doney et al., 2012; Smale et al., 2019). Again, impacts from extreme temperature anomalies on small marine animals have received little research attention (Chen et al., 2021b).

In near‐shore marine ecosystems, small animals that live on and around the surfaces of physical structures, i.e., benthic ‘epifauna’, are among the most common organisms that are facilitated by foundation species (Chen et al. 2021b; Kelaher, 2003, 2005; Matias et al., 2010). Typically, epifauna are small, fast‐growing mobile (or sometimes sessile) animals that rely on physical structures to avoid environmental stress and predators (Chen et al., 2021b). Epifauna also provide crucial trophic linkages between primary producers and higher‐order consumers, like larger crustaceans and small fish, making epifauna an essential component in marine food webs (Chen et al., 2021b). Laboratory and mesocosm experiments are powerful tools to test how epifauna respond to changing temperatures, as temperatures in closed systems can be controlled with high precision (Pansch et al., 2018; Truong et al., 2020). However, to understand and predict epibiota responses to future MHWs, manipulative field experiments are vital to bridge the precise laboratory and mesocosm experiments and non‐controlled before‐after MHW comparisons (Osman et al., 2010; Powell et al., 2019). Unfortunately, it is extremely difficult to manipulate oceanic temperature under open field conditions, and few field experiments have therefore tested how epifauna respond to MHWs in natural coastal ecosystems (none were reported in a recent comprehensive review of epifauna studies, Chen et al., 2021b). To address this research gap, we here provide a first test of how controlled and manipulated MHWs affect short‐term colonisation and abundances of epifauna onto co‐occurring mimics of seaweed turf (primary foundation species) and seaweed holdfast (secondary foundation species) attached to heated plates, under realistic open oceanic field conditions. Non‐living mimics of different morphological complexities were used to model morphological and structural effects from foundation species, similar to that done in many other studies that have tested ecological theories on epifaunal communities (Edgar & Klumpp, 2003; Kelaher, 2003, 2005; Matias et al., 2011; Navarro‐Barranco et al., 2022). Indeed, mimics are often used to test ecological theories because the texture, morphology, isolation, and successional stage of the experimental units can be controlled and manipulated, and because structurally robust mimics can be transplanted to specific environments and collected again with minimal loss (Hauser et al., 2006; Myers & Southgate, 1980; Smith & Rule, 2002; Thomsen et al., 2022). Epifaunal communities that colonise mimics typically mirror, albeit sometimes in lower abundances, the colonisation of the mimic's natural counterparts (Kelaher, 2003, 2005; Matias et al., 2011; Myers & Southgate, 1980; Thomsen et al., 2022).

More specifically, to address the outlined research gaps, we tested, in a four‐factorial field experiment, if colonisation and abundances of epifauna onto plates were affected by (a) 3–5°C heating, mirroring a recent extreme MHW in the study area, (b) the presence of secondary foundation species of increasing morphological complexities, and if effects varied between four trials done (c) within and (d) between summer and winter seasons, where each trial had different ambient oceanic temperatures (but only slightly different between the two summer trials). This approach allowed us to rank these test factors according to their ecological importance (using sum of squares as proxies), and test for possible additive, synergistic or antagonistic interactions between the MHW, the morphological complexity of the secondary foundation species, and the intra and inter‐seasonal conditions. We hypothesised that colonisation and abundances of epifauna by seaweed foundation species would be higher under heated conditions and warmer ambient conditions because elevated temperature can stimulate dispersal, recruitment, and growth of epifauna in cold‐temperate ecosystems (Dolbeth et al., 2012; Ledet et al., 2021). We also hypothesised that abundances of epifauna would be higher on plates with secondary foundation species, particularly when the secondary foundation species were morphologically more complex (using surface to planform area ratios of the seaweed holdfasts as a proxy), because complex habitats typically have more interstitial spaces and microhabitats that small animals can colonise and occupy (Thomsen et al., 2022; Tokeshi & Arakaki, 2012).

## 2 METHODS

### 2.1 Study area

The field experiment was done in Lyttelton harbour, Canterbury, New Zealand (172.712, −43.605). The salinity in Lyttelton harbour is fully marine year‐round (i.e., 35 psu), whereas sea surface temperature (SST) varies seasonally between 9 and 20°C. However, during a recent extreme MHW (the Tasman Sea 2017/18 MHW between 14 November 2017 and 9 April 2018), SST increased to more than 23°C. This MHW was the strongest event recorded over 38 years of existing satellite data and devasted local populations of endemic and native fucoid seaweed (Thomsen et al., 2019). The Tasman Sea MHW was categorised as an ‘extreme’ event, because the sea surface temperature exceeded 4× the 90th percentile threshold, relative to the local climatology, for more than five consecutive days (Hobday et al., 2016, 2018). Here, we manipulated and raised temperature by >3° in all our field trials to test how mobile epifauna respond to extreme temperature increases, as they would have experienced during the recent Tasman Sea 2017/18 MHW.

### 2.2 Heated plates and turf‐mimics

**FIGURE 1:**
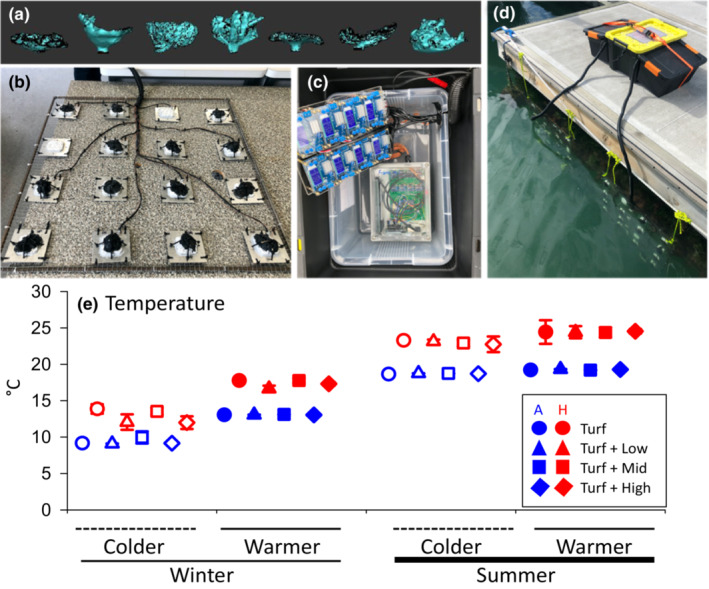
(a) Models of seaweed holdfast (*Durvillaea antarctica*, *D. poha*, *Macrocystis pyrifera*, *Ecklonia radiata*, *D. willana*, *Undaria pinnatifida,* and *Carpophyllum maschalocarpum*). (b) Experimental frame containing eight heated and eight control plates (*n* = 16 per frame), with combinations of turf (white) and turf + holdfast mimics (white and black) secured to plates with different heatwave treatments. (c) Adjustable regulator per Peltier device (heated temperature treatment) and Arduino Uno with thermocouples for logging temperature (black and orange box Figure 1d). (d) Frames deployed on buoy with shore‐based 100 amp per hour battery (yellow box). (e) Mean values (±SE) of mean temperature per plate across heating (ambient vs. heated), seasons (summer vs. winter), intra‐seasons (cold vs. warm), and morphological complexity of added secondary foundation species. *N* for turf alone, Turf + Low, Turf + Mid, Turf + High habitat complexity = 2, 4, 6, 4, respectively.

**FIGURE 2:**
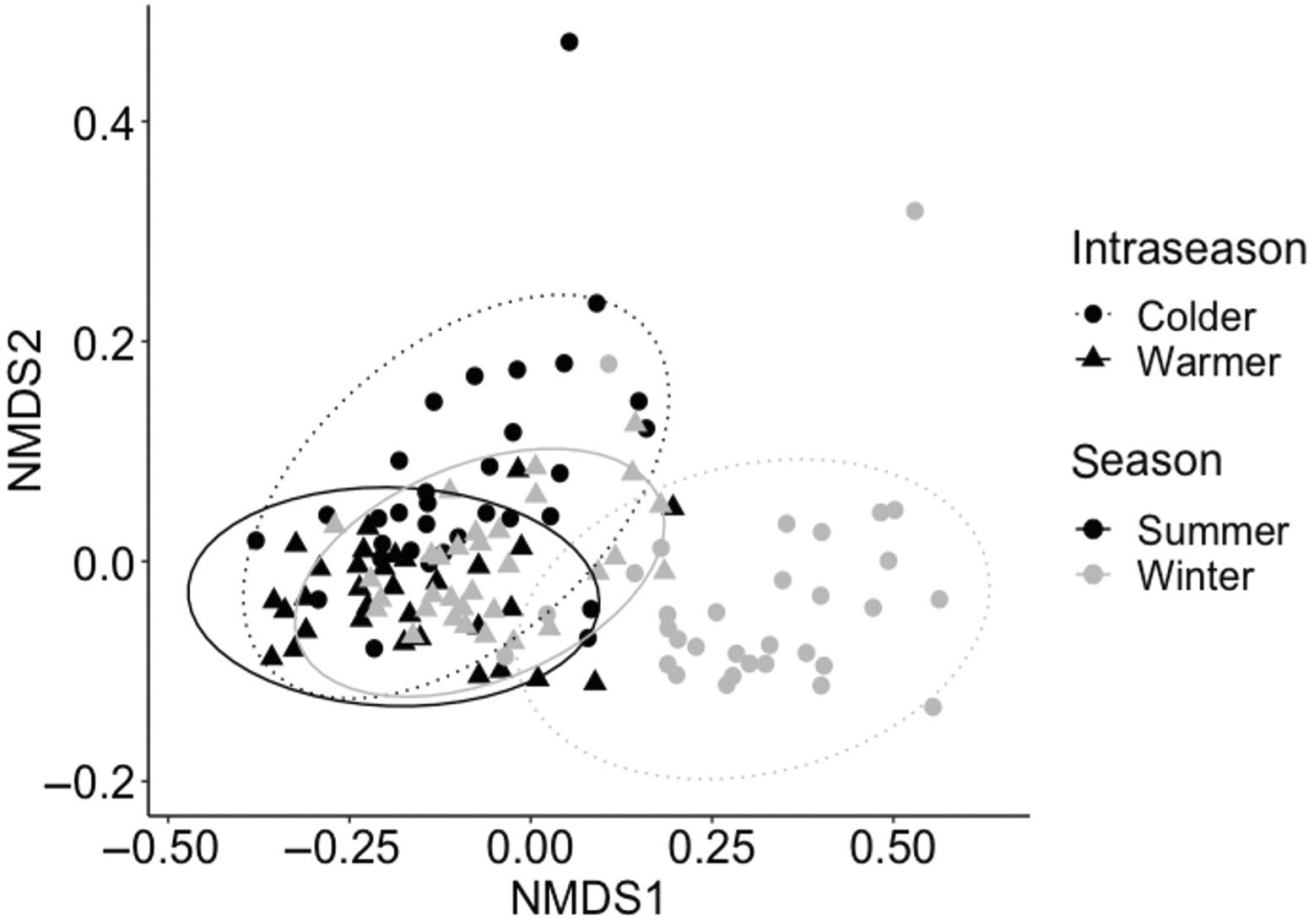
Multidimensional scaling (NMDS) plot showing similarities in animal communities analysed on operational taxonomic units (OTU) coded for significant interaction between season (summer = black, solid ellipses and winter = grey, dash ellipses) and intra‐season (cold = circle, solid ellipses and warm = triangle, dashed ellipses). Distances were calculated with Bray‐Curtis's dissimilarity metrics on epifauna count data. *N* = 128.

To measure the colonisation and abundances of epifauna, we constructed thirty‐two 100 cm^2^ aluminium plates, like those used in past experiments testing for heating impacts on sessile marine species (Kordas et al., 2015; LaScala‐Gruenewald & Denny, 2020; Lathlean et al., 2017; Smale et al., 2011). Sixteen of the plates were fitted with a 25 cm^2^ Thermoelectric Peltier device in the centre, and sixteen were fitted with control devices that did not omit heat. Peltier devices were secured to the aluminium settlement plate and covered in a layer of waterproofing silicon which allowed effective heat transfer from the Peltier device. The top layer of silicon was manipulated using a wire brush to create texture and three‐dimensional habitat that mimicked the morphology and rugosity of natural turf seaweed as best as possible (Figure 1). Power for heating was supplied to each Peltier device via a cable connected to a shore‐based 100 amp/h rechargeable car battery and energy input was individually programmed and maintained between 1.25 and 1.35 watts using adjustable regulator devices (Figure 1). This energy input heated the water in the boundary layer in‐between and just above the turf filaments (i.e., on plates without holdfast) and in‐between the turf and seaweed holdfasts (see the next section) by 3–5°C (Figure 2). The heating period, i.e., the duration of each of the four trials (see Section 2.4) lasted for a week (seven nights, eight days), and was checked daily to monitor energy input (watts) for individual Peltier devices and to change batteries when needed.

### 2.3 Holdfast mimics

Holdfast mimics were 3D printed and modelled seven canopy‐forming seaweed species of varying morphological complexity, including three New Zealand endemic fucoids (*Carpophyllum maschalocarpum*, *Durvillaea poha*, and *D. willana*), three widely distributed fucoids and kelps (*D. antarctica*, *Ecklonia radiata*, and *Macrocystis pyrifera*) and the non‐native Japanese kelp (*Undaria pinnatifida*) that have invaded the lowest part of the intertidal rocky zone in many places in New Zealand (South et al., 2017; Thomsen & South, 2019). All seven species are common in the wider region and can form dense stands with numerous holdfasts and extensive canopies. Mimics of *U. pinnatifida* and *D. poha* were printed from existing 3D models as described in Thomsen et al. (2018a). These two models were made using photogrammetry, from 61 photographs taken from different angles of an 8 cm diameter live holdfast and converted to 3D models in Autodesk Memento. The remaining five holdfast mimics were digitised by scanning fresh holdfasts with a Kreon Arm Probe, whereafter 3D models were stitched together in Geo Magic Wrap (Artec 3D, 2017). All holdfast mimics were scaled to similar sizes, i.e., with a maximum length of 60–73 mm, without morphological distortion and printed in a rubbery and flexible biocompatible filament (Figure 4 RUBBER‐65A BLK) at Fi Innovations, New Zealand (Figure 1a,b). This rubbery filament had a similar texture to live holdfasts.

### 2.4 Field experiment

The colonisation experiment was done on a total of 128 independent plates (with attached seaweed mimics) covering the following fully crossed test factors: 2 heating treatments (heating vs. no heating controls) × 4 mimic‐complexities (turf‐alone vs. turf with attached holdfast of low, medium and high complexity) × 2 seasonal trials (winter vs. summer) × 2 within‐seasonal trials (with relatively warm vs. cold intra‐seasonal ambient water temperature) × 2–6 replicates (with higher replication level for specific holdfast complexity treatments, see legend in Figure 2). For each of the four trials, two 4.9 m^2^ metal frames (each frame with 16 plates) were deployed on floating pontoons in Te Ana Marina, Lyttelton Harbour, hanging vertically 30 cm below the sea surface to avoid sediment accumulations. To ensure a stable vertical profile, weights were attached to the bottom of each frame. The positioning on floating pontoons implied that the plates were always submerged, and colonising animals therefore did not experience desiccation stress. The two frames were deployed two times during the austral summer of 2022 (15–22/2 with average SST = 19.3°C) vs. (24/2 to 2/3 with average SST = 18.6°C) and two times during the winter of 2022 (18–24/5 with average SST = 13.0°C vs. 21–28/6 with average SST = 9.1°C; Figure S1). The 16 plates on each frame were evenly spaced 10 cm apart and secured by cable tie for easy retrieval (Figure 1). Each metal frame had a heated and no‐heated eight‐plate arrays, covered by a turf‐only (*N* = 4) and seven turf + holdfast combinations (*N* = 28, see Figure 2 for replication levels for the low, medium, and high holdfast complexities). Holdfast mimics were placed randomly on the metal frame and secured onto the turf by two cable ties (also added to the turf‐alone plates) and fitted to cover the Peltier device. Frames were deployed for each trial on the same pontoons to keep environmental conditions, like water flow and turbidity, consistent.

Water temperature was measured on heated plates every 2 s for the entire duration of each trial, by inserting 0.1 mm K‐type thermocouples a few mm above the turf (for plates with no holdfast) or in the interstitial space between turf and holdfasts. Measurements were saved to a microSD card using an Arduino Uno with 16 MAX31855 thermocouple amplifiers and a DS3231 real time clock (Figure 1). The ambient water temperature around the non‐heated control plates was also measured every 2 s using 16 Onset HOBO data loggers placed beside each of control plates.

Upon collection, cable ties were removed, and each individual plate and their mimics were secured in zip lock bags to avoid loss of epifauna. Zip lock bags with mimics and epifauna were transported to the laboratory and rinsed over a 250 μm sieve. Unfortunately we were unable to identify epifauna to species level but instead recorded higher taxonomic units (like class or family) as well as lower level morphologically determined ‘operational taxonomic units’ (OTUs; morphotypes, as in Montie & Thomsen, 2023, Thomsen et al., 2022). Morphotypes were identified within the classes hexanauplia, nematoda, polychaeta, and gastropoda. Furthermore, for the malacostraca class, we also identified the caprellidae family, and morphotypes within the amphipoda, decapoda, and isopoda orders. It is possible that different morphotypes were the same species (implying that sample richness is over‐estimated) although it is more likely that species were overlooked, in particular for abundant cryptic copepods (implying that sample richness is under‐estimated). The less common epifauna, here grouped into remaining ‘other taxa’, included the classes ostracoda, maxillopoda, foraminifera, and hexacorallia. Although the OTU approach is not identical to an analysis of species richness, statistical results done at a higher‐class level were very similar to the OTU analyses (see next section) suggesting our results are robust. Furthermore, the OTU approach does not affect statistical analyses of abundances.

### 2.5 Statistical analysis

Fixed four‐factorial PERMANOVAs were used to analyse for patterns in multivariate community structure (using Bray–Curtis dissimilarity on square root transformed abundances to downplay the importance of dominant taxa), and univariate responses. The univariate responses included temperature, epifaunal taxonomic richness, total epifaunal abundances, and abundances of dominant epifaunal classes, i.e., hexanauplia and malacostraca, as well as abundances of remaining ‘other’ taxa. The univariate responses were square root transformed (or fourth root for ‘other’ taxa) to remove variance heterogeneity (*p* > .14 for all Levene's overall tests). The fixed factors included MHW treatment (heated vs. ambient control), morphological complexity of mimics (turf alone vs. turf with holdfasts of low, medium, or high complexity), season (summer vs. winter), and intra‐season (colder vs. warmer ambient temperatures recorded during the experimental trails within each season). The trials done within a season were treated as a fixed test factor that, from the measured temperature data, were reclassified as the ‘warmer’ or ‘colder’ summer (or winter) trial (see Figure 1 for specific temperatures measured within and between seasons). The morphological complexity of the holdfasts was calculated as the ratio (Sa:Pa) between a species total surface area (‘wetted’ area that include crack and crevices, in mm^2^) to planform area (area looking down perpendicular on the holdfasts, in mm^2^). These metrics were measured and calculated from horizontal splicing of the 3D models, which removed the seaweed stipe from some holdfast mimics (Figure 1). Based on the Sa:Pa ratios, holdfasts were reclassified as low (*D. antarctica*, *D. willana*), medium (*C. maschalocarpum*, *E. radiata*, *M. pyrifera*), or high (*D. poha*, *U. pinnatifida*) morphological complexity (Table 1). This morphological reclassification resulted in two, four, six, and four replicates for turf alone and turf with holdfasts of low, medium, or high complexity, respectively, for both heated and control plates and for each of the four trials. Significant contrasts for the holdfast complexity test factor were identified with SNK post hoc tests (Table S2, Figure S2). The holdfast size attributes were correlated against epifaunal univariate responses (Spearman's rank coefficient, Table S1, where holdfast sizes = length, width, surface area, and planform area), and the lack of significant effects demonstrated that the amount of holdfast habitat did not confound the analysis of complexity effects. Furthermore, the multivariate community structure was visualised with a nMDS plot and significant single factor effects where analysed with Similarity of Percentages (SIMPER) to identify taxa that contributed most to community differences. To test if the different taxonomic resolutions (OTUs) used for different taxa affected our results, analysis of community and diversity metrics were repeated on the higher‐class level. Results were very similar between the OTU and class‐level analyses, so only the former results are shown here.

**TABLE 1:** Key attributes for seven seaweed holdfasts, including maximum length (L, in mm), maximum width (W, in mm), surface area (Sa, in mm^2^), planform area (Pa, in mm^2^), surface area to planform area (Sa:Pa) and their complexity groping based on the Sa:Pa ratios.

Species	L (mm)	W (mm)	Sa (mm^2^)	Pa (mm^2^)	Sa:Pa	Grouping
*Durvillaea poha*	74	48	9719	2248	4.32	High
*Undaria pinnatifida*	73	47	9882	2392	4.13	High
*Macrocystis pyrifera*	73	43	8983	2330	3.85	Medium
*C. maschalocarpum*	60	38	7437	1941	3.83	Medium
*Ecklonia radiata*	64	52	8271	2307	3.59	Medium
*Durvillaea antarctica*	65	44	6819	2320	2.94	Low
*Durvillaea willana*	60	59	6991	2515	2.78	Low

## 3 RESULTS

### 3.1 Temperature

The full statistical model for temperature explained 98% of the total data‐variability (Sum of Squares, hereafter SS). The temperature on the plates was significantly affected by season × intra‐season × heating (*p* < .001), intra‐season × heating × complexity (*p* = .013), and season × intra‐season (*p* < .001, 2% of SS). When pooling across test‐factors, season (summer vs. winter; *p* < .001, 61%), intra‐season (colder vs. warmer temperatures during experimental trails within each season; *p* < .001, 7%) and heating (heated vs. ambient controls) were also significant (*p* < .001, 26%; Figure 1e, Figure S3, Table 2a).

**TABLE 2:** (a–f) Fixed four‐factorial Permutational multivariate analysis of variance on operational taxonomic units of animals, using Bray–Curtis dissimilarity coefficients for multivariate data and Euclidian distance for univariate responses for temperature (a), multivariate community structure (b), taxonomic richness (c), and abundances of all individuals (d), hexanauplia (e, copepods), malacostraca (f, mainly amphipods), and ‘other’ class taxa (g).

	df	SS	%	*F*	*p*
a. Temperature					
Season	1	66.36	61	5825.468	**<.001**
Intra‐season	1	7.59	7	666.518	**<.001**
Heatwave	1	28.57	26	2508.282	**<.001**
Complexity (morphology)	3	0.05	0	1.524	.209
Season × Intra‐season	1	2.31	2	203.183	**<.001**
Season × Heatwave	1	0.02	0	2.033	.155
Intra‐season × Heatwave	1	0.03	0	2.622	.107
Season × Complexity	3	0.01	0	0.19	.903
Intra‐season × Complexity	3	0.11	0	3.11	**.027**
Heatwave × Complexity	3	0.05	0	1.431	.235
Season × Intra‐season × Heatwave	1	0.38	0	33.723	**<.001**
Season × Intra‐season × Complexity	3	0.04	0	1.173	.321
Season × Heatwave × Complexity	3	0.01	0	0.19	.903
Intra‐season × Heatwave × Complexity	3	0.13	0	3.683	**.013**
Season × Intra‐season × Heatwave × Complexity	3	0.04	0	1.283	.281
Residuals	96	2.55	2		
b. Community structure					
Season	1	3.7419	18	44.716	**.001**
Intra‐season	1	3.1331	15	37.441	**.001**
Heatwave	1	0.2807	1	3.354	**.017**
Complexity (morphology)	3	0.8114	4	3.232	**.001**
Season × Intra‐season	1	2.1164	10	25.29	**.001**
Season × Heatwave	1	0.1305	1	1.559	.174
Intra‐season × Heatwave	1	0.1025	1	1.225	.257
Season × Complexity	3	0.3624	2	1.444	.152
Intra‐season × Complexity	3	0.4552	2	1.813	(.089)
Heatwave × Complexity	3	0.2303	1	0.917	.501
Season × Intra‐season × Heatwave	1	0.1392	1	1.664	.155
Season × Intra‐season × Complexity	3	0.1427	1	0.568	.850
Season × Heatwave × Complexity	3	0.3042	1	1.212	.281
Intra‐season × Heatwave × Complexity	3	0.2839	1	1.131	.303
Season × Intra‐season × Heatwave × Complexity	3	0.2271	1	0.905	.513
Residuals	96	8.0336	39		
c. Taxonomic richness					
Season	1	11.933	32	101.44	**<.001**
Intra‐season	1	6.126	16	52.075	**<.001**
Heatwave	1	0.518	1	4.399	**.039**
Complexity (morphology)	3	0.732	2	2.075	.101
Season × Intra‐season	1	3.324	9	28.253	**<.001**
Season × Heatwave	1	0	0	0.004	.953
Intra‐season × Heatwave	1	0.063	0	0.538	.465
Season × Complexity	3	0.281	1	0.797	.498
Intra‐season × Complexity	3	0.279	1	0.791	.502
Heatwave × Complexity	3	0.023	0	0.066	.978
Season × Intra‐season × Heatwave	1	0.002	0	0.018	.892
Season × Intra‐season × Complexity	3	0.071	0	0.202	.895
Season × Heatwave × Complexity	3	1.320	3	3.741	**.014**
Intra‐season × Heatwave × Complexity	3	1.253	3	3.551	**.017**
Season × Intra‐season × Heatwave × Complexity	3	0.574	2	1.626	.188
Residuals	96	11.293	30		
d. Abundance all epifauna					
Season	1	466.5	36	126.042	**<.001**
Intra‐season	1	227.5	18	61.48	**<.001**
Heatwave	1	33.5	3	9.041	**.003**
Complexity (morphology)	3	57.7	4	5.193	**.002**
Season × Intra‐season	1	84	7	22.705	**<.001**
Season × Heatwave	1	1.9	0	0.514	.475
Intra‐season × Heatwave	1	0	0	0.012	.912
Season × Complexity	3	25.6	2	2.302	.082
Intra‐season × Complexity	3	8.7	1	0.784	.506
Heatwave × Complexity	3	7.6	1	0.683	.564
Season × Intra‐season × Heatwave	1	0	0	0.003	.960
Season × Intra‐season × Complexity	3	6.5	1	0.583	.628
Season × Heatwave × Complexity	3	5.9	0	0.529	.663
Intra‐season × Heatwave × Complexity	3	2.4	0	0.212	.888
Season × Intra‐season × Heatwave × Complexity	3	3.2	0	0.287	.835
Residuals	96	355.3	28		
e. Abundance hexanauplia					
Season	1	312.1	26	77.001	**<.001**
Intra‐season	1	279.9	23	69.066	**<.001**
Heatwave	1	37.5	3	9.26	**.003**
Complexity (morphology)	3	50.2	4	4.131	**.008**
Season × Intra‐season	1	24.2	2	5.967	**.016**
Season × Heatwave	1	21.2	2	5.227	**.024**
Intra‐season × Heatwave	1	2.8	0	0.702	.404
Season × Complexity	3	38.6	3	3.172	**.028**
Intra‐season × Complexity	3	2.1	0	0.169	.917
Heatwave × Complexity	3	12.8	1	1.054	.373
Season × Intra‐season × Heatwave	1	4.4	0	1.096	.298
Season × Intra‐season × Complexity	3	8.2	1	0.674	.570
Season × Heatwave × Complexity	3	8	1	0.655	.581
Intra‐season × Heatwave × Complexity	3	18.4	2	1.515	.216
Season × Intra‐season × Heatwave × Complexity	3	6.9	1	0.564	.640
Residuals	96	389	32		
f. Abundance malacostraca					
Season	1	150.61	37	118.91	**<.001**
Intra‐season	1	20.04	5	15.825	**<.001**
Heatwave	1	1	0	0.791	.376
Complexity (morphology)	3	5.19	1	1.365	.258
Season × Intra‐season	1	77.57	19	61.246	**<.001**
Season × Heatwave	1	0.92	0	0.73	.395
Intra‐season × Heatwave	1	0.46	0	0.366	.546
Season × Complexity	3	2.42	1	0.637	.593
Intra‐season × Complexity	3	0.79	0	0.208	.890
Heatwave × Complexity	3	0.09	0	0.025	.995
Season × Intra‐season × Heatwave	1	3.96	1	3.123	(.080)
Season × Intra‐season × Complexity	3	9.86	2	2.595	(.057)
Season × Heatwave × Complexity	3	1.62	0	0.426	.735
Intra‐season × Heatwave × Complexity	3	10.71	3	2.817	**.043**
Season × Intra‐season × Heatwave × Complexity	3	1.61	0	0.423	.737
Residuals	96	121.59	30		
g. Abundance ‘other’ class taxa					
Season	1	0.79	1	2.287	.134
Intra‐season	1	0.01	0	0.023	.879
Heatwave	1	0.17	0	0.485	.488
Complexity (morphology)	3	0.75	1	0.725	.539
Season × Intra‐season	1	4.27	8	12.42	**.001**
Season × Heatwave	1	0.06	0	0.187	.667
Intra‐season × Heatwave	1	0.64	1	1.867	.175
Season × Complexity	3	2.33	4	2.258	(.087)
Intra‐season × Complexity	3	2.12	4	2.057	.111
Heatwave × Complexity	3	0.8	1	0.777	.510
Season × Intra‐season × Heatwave	1	0.14	0	0.412	.522
Season × Intra‐season × Complexity	3	0.17	0	0.163	.921
Season × Heatwave × Complexity	3	0.64	1	0.622	.602
Intra‐season × Heatwave × Complexity	3	5.03	9	4.868	**.003**
Season × Intra‐season × Heatwave × Complexity	3	3.68	7	3.569	**.017**
Residuals	96	33.04	60		

*Note*: All factors were considered fixed including season (summer, winter), intra‐season (warm, cold), heatwave (heated, ambient), and morphological habitat complexity (no holdfast, low, medium, high). Significant *p*‐values (*α* < .05) are shown on bold and *p*‐values <.1 are shown in brackets.

### 3.2 Community structure

A total of 8403 epifauna, all in the 250–5000 μm size range, colonised the 128 plates across the four trials. The general epifaunal community was dominated by copepods (class hexanauplia; 75.4%), and amphipods (class malacostraca; 19.1%), whereas ‘other’ taxa such as bivalves, gastropods, ostracods, and polychaetes together represented 5.3% of the community. Test factors explained in concert 61% of the total data‐variability. Epifauna community structure was significantly affected by season × intra‐season (*p*_community_ = .001, 10% of SS) and the four individual test factors (in order of importance: *p*_season_ = .001, 18%, *p*_intra‐season_ = .001, 15%, *P*_complexity_ = .001, 4%, *P*_heating_ = .017, 1%; Figure 2, Table 2b). The main taxa that explained differences between seasons and intra‐seasons included canthocamptid copepods (30% and 32% of the dissimilarity), a ‘white’ Gammaridae amphipod morphotype (16% and 15%), and a ‘brown spotted’ Gammaridae amphipod morphotype (10% and 9%). Canthocamptid copepods were also most important to separate differences between heating treatments (30%), followed by the ‘white’ Gammaridae (16%), and cyprid barnacles (9%). Furthermore, canthocamptid copepods were most important to separate holdfast complexity treatments (27%–33% across the test‐combinations) again followed by ‘white’ Gammaridae (14%–16% across combinations).

### 3.3 Taxonomic richness and abundances

**FIGURE 3:**
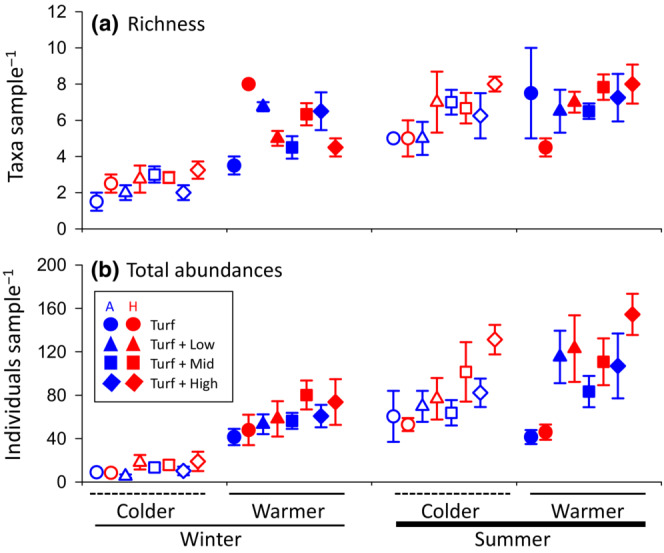
Mean values (±SE) of taxonomic richness (a) and abundances (b) of animals per turf‐mimic across heating (ambient vs. heated), seasons (summer vs. winter), intra‐seasons (cold vs. warm), and morphological complexity of added secondary habitat‐formers. *N* for turf alone, Turf + Low, Turf + Mid, Turf + High habitat complexity = 2, 4, 6, 4, respectively.

The full statistical model explained 70% of the richness response, with several significant interactions, including season × heating × complexity (*p* = .014, 3% of SS), intra‐season × heating × complexity (*p* = .017, 3%), and season × intra‐season (*p* = .001, 9%). Furthermore, season (*p* < .001, 32%), intra‐season (*p* < .001, 16%) and heating were significant single factors (*p* = .039, 1%, Figure 3a, Table 2c). Taxonomic richness was highest on heated plates with high holdfast complexity collected during the warmer summer trial (8.00 ± 0.00 taxa per plate) and lowest on control plates with no holdfast (turf only) collected during the colder winter trial (1.50 ± 0.50). On average and pooled across other test factors, taxonomic richness was higher in summer compared to winter (6.75 ± 0.26 vs. 4.09 ± 0.25), during warmer intra‐seasonal trials compared to the cooler trials (6.31 ± 0.32 vs. 4.53 ± 0.24), on heated compared to the ambient control plates (5.63 ± 0.30 vs. 5.17 ± 0.31), and increased along the holdfast complexity gradient (5.72 ± 0.46 in the highest complexity habitat vs. 4.69 ± 0.64 in the turf only habitat).

For total epifaunal abundances, the full model explained 72% of the data‐variability. Epifaunal abundance was significantly affected by season × intra‐season (*p* < .001, 7% of SS) and the four individual test factors (in order of importance: *p*_season_ < .001, 36%, *p*_intra‐season_ < .001, 16%, *P*_complexity_ = .002, 4%, *P*_heating_ = .003, 3%, Figure 3b, Table 2d). Total epifaunal abundance was highest on heated plates with high holdfast complexity collected during the warmer summer trial (154.5 ± 18.95 taxa per plate) and lowest on plates without heating collected during winter trials and with low holdfast complexity (5.50 ± 1.5). On average, total abundance was generally higher in summer than winter (93.72 ± 6.03 vs. 37.58 ± 3.98), for the warmer intra‐seasonal trials (83.14 ± 5.84 vs. 48.16 ± 5.84 for the colder intra‐seasonal trials), on heated plates (74.78 ± 6.93 vs. 56.45 ± 5.17 for ambient temperatures), and increased along the holdfast complexity gradient (38.50 ± 5.45 on turf‐alone plates vs. 79.84 ± 9.97 on the turf + high complexity plates). Specifically, SNK post hoc tests showed that epifaunal abundances associated with the turf‐only plates were significantly lower than on plates with turf + low (*p* < .001), turf + mid (*p* = .0065) and turf + high (*p* = .0057) holdfast complexity.

**FIGURE 4:**
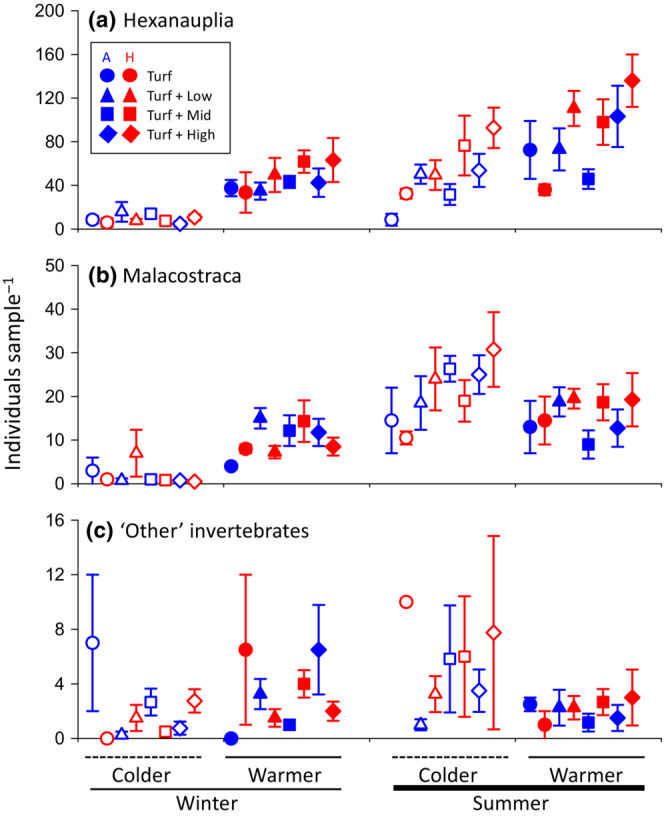
Mean abundances (±SE, *N* = 4) of hexanauplia (copepods) (a), malacostraca (mainly amphipods) (b) and ‘other’ invertebrate animals (c) per turf‐mimic across temperatures (control vs. heated), seasons (summer vs. winter), intra‐seasonal temperatures (cold vs. warm), and morphological complexity of added secondary habitat‐former. The outlier result for ‘Other invertebrates’ inhabiting ambient colder summer turf alone mimics were removed for clarity (37.5 ± 21.5). *N* for turf alone, Turf + Low, Turf + Mid, Turf + High habitat complexity = 2, 4, 6, 4, respectively.

Hexanauplia dominated the epifauna across samples, and statistical tests results were therefore relatively similar to the analysis for total abundances. Specifically, 68% of the data variability was explained by the full statistical model with significant effect of season × intra‐season (*p* = .016, 2%), season × heating (*p* = .024, 2%), and season × complexity (*p* = .028, 3%) and all four individual test factors (in order of importance: *p*_season_ < .001, 26%, *p*_intra‐season_ < .001, 23%, *P*_complexity_ = .008, 4%, *P*_heating_ = .003, 3%, Figure 4a, Table 2e). The abundance of hexanauplia were highest on the heated plates with high holdfast complexity, during the warmer summer trial (136.00 ± 23.98 taxa per plate) and lowest on ambient control plates during the colder winter trial (4.75 ± 2.50). On average, abundances of hexanauplia were higher on plates collected in summer (70.11 ± 5.91 vs. 28.84 ± 3.32 in winter), in the warmer intra‐seasonal trial (67.23 ± 5.31 vs. 31.72 ± 4.61 in the cold intra‐season trial), on heated plates (58.73 ± 6.29 vs. 40.21 ± 4.15 on ambient control plates), and increased along the holdfast complexity gradient (29.37 ± 6.20 on turf‐alone plates vs. 63.38 ± 9.43 in the turf + high holdfast complexity). The SNK post hoc test showed that hexanauplia were significantly less abundant on turf‐only plates compared to plates with turf + mid (*p* = .0358) and turf + high (*p* < .001) holdfasts complexities.

For abundances of malacostraca, the significant test factors explained, in concert, 70% of the data variability. Abundances of malacostraca were significantly affected by intra‐season × heating × complexity (*p* = .043, 3%), season × intra‐season (*p* < .001, 19%) as well as season (*p* < .001, 37%) and intra‐season (*p* < .001, 5%). More specifically, malacostraca were most abundant on heated plates with high holdfast complexity, collected during colder summer trials (30.75 ± 8.55) and least abundant on heated plates with high holdfast complexity collected during colder winter trials (0.50 ± 0.29) (Figure 4b, Table 2f). Finally, the test factors for remaining ‘Other’ taxa, explained, in concert, only 40% of the data variability. Here, only the full four‐way interaction (*p* = .017, 7%), intra‐season × heating × complexity (*p* = .034, 9%) and season × intra‐season (*p* = .001, 8%) were significant. ‘Other’ taxa were generally most abundant on heated plates with turf‐only collected in the colder summer trial (10.00 ± 0.00) and least abundant on the non‐heated control plates with turf‐only collected during both the colder and warmer winter trials (both with zero animals, Figure 4c, Table 2g).

## 4 DISCUSSION

Co‐occurring foundation species can facilitate animals by providing living space, ameliorate stress, and reduce consumer pressure, but little is known about how these facilitated animals are affected by human stressors, like strong heatwaves, by the morphological complexity of the foundation species themselves, or by seasonal changes in abiotic conditions. Here, we showed that week‐long colonisation of small mobile animals (epifauna) onto mimics of foundation species were greatest during simulated heatwaves, in summer and warmer intra‐seasonal trials, and in the presence of secondary foundation species, particularly when the secondary foundation species was morphologically complex. Furthermore, several statistical interactions were important, demonstrating that the magnitude of effects varied depending on the spatiotemporal context, e.g., showing that abundances (i.e., facilitation) was stronger in the slightly warmer intra‐season during summer.

### 4.1 Heating

Climate changes and stronger heatwaves are changing ecological communities, as cold‐water adapted species reach critical thermal limits (Smale et al., 2017; Smith et al., 2023; Wernberg et al., 2011, 2016). Several studies have shown how changing ocean conditions can affect epifauna (Chen et al., 2021b; Ratnarajah et al., 2023), e.g., by quantifying responses to heatwaves from before‐after field observations (Chen et al., 2021a), in laboratory and mesocosm experiments (Pansch et al., 2018; Truong et al., 2020), and in field experiments (Ashton et al., 2017; Clark et al., 2019; Smale et al., 2011). However, these field experiments were designed to test for impact of heatwaves on the colonisation of sessile species within a narrow (mm thick) fluid boundary layer. Results from these studies suggest that temperate epifauna tolerate or are facilitated by heatwaves. Our results supported these findings, because richness and total abundances of epifauna were higher on heated plates, although this pattern was largely driven by copepods which made up c. 75% of the community. Note however, that heating effects explained less data variability than the other significant test factors, probably because each trial, i.e., duration of heating, only ran for 1 week. The dominant copepods are small ubiquitous seaweed‐associated epifauna with short life histories and efficient dispersal through the water column (Hall & Bell, 1988, 1993; Sasaki & Dam, 2021), probably explaining why these organisms were most abundant on our plates and why they were facilitated by the MHW treatment. Still, copepods from tropical systems could, in contrast to our results, be inhibited by MHWs that at lower latitudes would be superimposed on warmer ambient temperatures, and likely be closer to specific copepod species upper thermal limits (Truong et al., 2020). Altogether, our experimental findings align with global reviews over warming tolerances (i.e., difference between thermal optimum and maximum annual temperature), that also suggest that copepods from cooler high latitudes are less vulnerable to extreme temperatures, compared to copepods in warmer low latitudes (Sasaki & Dam, 2021). In contrast to the copepod results, we did not find significantly more malacostraca (dominated by gammarid amphipods) on the heated plates. However, amphipod abundances were both less abundant and more variable, making it difficult to detect significant heating effects. Amphipods are relatively fast colonisers of seaweeds (although slower than copepods), and often have wide temperature tolerances, as the same species can be found associated with seaweed in both warmer summer and colder winter months (Tsoi et al., 2005; Viejo, 1999; Wernberg et al., 2004). Indeed, laboratory experiments have shown that amphipods inhabiting temperate *Sargassum* seaweed in Australia are largely unaffected by experimental heatwaves (Ledet et al., 2021), and that survival and mobility only decreased when temperature far exceeded near‐future projections of MHWs. Furthermore, greater thermal tolerance has been found for amphipods collected in summer compared to winter (i.e., for warm acclimatised individuals), suggesting that MHW impacts on temperate amphipods may depend on local species composition and timing relative to recent climatic conditions (Ledet et al., 2021). These experiments were, however, done over very short time scales (hours) and, like our 1‐week experiment, did not consider longer time lag‐effects. For example, exposure to high temperature can slowly deplete nutrient reserves and reduce metabolic capacities, so that population‐wide effects only effectuate on longer time scales (Filbee‐Dexter et al., 2020). This has been shown for seaweed foundation species after simulated (Nepper‐Davidsen et al., 2020) and real (Wernberg et al., 2018) MHWs, but not yet for marine epifauna. Importantly, negative impacts from MHWs on seaweed foundation species are likely to also cause indirect lag effect on epifauna, as their critical habitat is lost and they become more susceptible to predation or environmental stress (Doney et al., 2012; Montie & Thomsen, 2023; Schiel et al., 2021; Smale et al., 2019).

### 4.2 Seasonality

Understanding how marine animal communities change within and between seasons is a fundamental research question (Chen et al., 2021b), where variabilities between seasons have been studied in some detail (Ba‐Akdah et al., 2016; Ledet et al., 2021; Leite & Turra, 2003; Taylor, 1997; Wernberg et al., 2004; Winkler et al., 2017). These studies highlight that season plays a major role in controlling epifauna community structure, probably because seasonality covaries with the sizes and morphology of the foundation species themselves (they are often bigger in summer) and changes to ambient environmental conditions, like temperature, day length, food availability, and wave action. Specifically, we found c. 2.75 more taxa and c. 59 more individuals per sample in the summer trials compared to the winter trials, supporting the above‐mentioned studies that typically shows more diverse and abundant epifauna in warmer seasons. While our results aligned with past seasonal epifauna research, it remains important to continue to test for season‐specific effects of MHWs since abundances of individual taxa can fluctuate widely throughout the year, because different epifaunal species can dominate in winter or summer (Taylor, 1997; Winkler et al., 2017). Furthermore, extreme temperatures, particularly during unusually hot summer months, may exceed the thermal optimum of the local epifaunal species, as shown for some tropical communities (Truong et al., 2020). We here complemented the many traditional seasonal epibiota studies by also showing ecological relevance of testing for intra‐seasonal effects, were the slightly warmer trials within a season (c. 1–3°C warmer than the colder intra‐seasonal trial) had higher richness and abundances of epibiota (c. 2.2 taxa and 35 individuals per sample, respectively). Given that the mimics themselves (in contrast to many live foundation species) did not vary in morphology or size within and between seasons, the high colonisation and abundances observed on plates in the warmer summer trials is likely associated with higher food availability, seasonally controlled ontogenetic life‐histories (like reproduction and water column dispersal), and faster movements and metabolism (like growth and reproduction) under high temperatures (Ba‐Akdah et al., 2016; Chen et al., 2021b; Ledet et al., 2021; Leite & Turra, 2003; Taylor, 1997; Wernberg et al., 2004; Winkler et al., 2017).

### 4.3 Complexity of foundation species

Seaweed turf and holdfasts provide complex structural habitats for animals, typically resulting in higher animal diversity compared to adjacent flat and simpler rock structures (Kelaher et al., 2001, 2003; Matias et al., 2010; Thomsen et al., 2018a; Tuya et al., 2011). Our results showed that richness and abundances of epifauna were higher on plates with co‐occurring turf and high holdfast complexity compared to plates with no holdfasts (or low holdfast complexity). Addition of the holdfasts, i.e., the secondary structure, to the turf mimics resulted in c. 40 more individuals on each plate, and even more animals under and around the most complex holdfasts. This result suggests that if canopy‐forming seaweed are lost, like after extreme MHWs (Montie & Thomsen, 2023; Smale et al., 2019), the biodiversity of epifauna and their ecological importance, like trophic transfers in food webs, will be significantly reduced. Many studies have shown similar positive effects from habitat complexity on epifaunal diversity and abundances (Hall & Bell, 1988; Martin‐Smith, 1993; Stoner & Lewis, 1985), probably because complexity covary with morphological attributes like surface area, fractal dimensions and interstitial spacing (Tokeshi & Arakaki, 2012), that can support more epifaunal species with different niches (Hauser et al., 2006; Hooper & Davenport, 2006; Veiga et al., 2014).

Although we documented more epifauna around mimics of complex foundation species, we did not test for specific underpinning facilitation mechanisms. Other studies have shown that epifaunal richness and abundance correlate with the composition and size of the foundation species (Davenport et al., 1996; Veiga et al., 2014), and their ecological function, like water retention and relative humidity in intertidal systems (Davenport et al., 1999). However, in our experiment, the composition and size of the mimic holdfasts was constant over time, allowing us to isolate structure itself as a driver of abundances (Thomsen et al., 2022). Furthermore, facilitation through stress‐amelioration is likely of low relevance, because the epifauna on the constantly submerged plates did not experience desiccation stress (or other obvious differences in environmental stress). We suggest that refugia from predation is the key facilitative process, because small mobile predators, like triplefin fish, blennies, and wrasses, are abundant in shallow subtidal systems, where they can exert strong top‐down control of epifauna in open unstructured habitats (Chen et al., 2021b; Gribben et al., 2019; Hall & Bell, 1988; Leite & Turra, 2003; Martin‐Smith, 1993; Stoner & Lewis, 1985). Future studies could test this hypothesis by crossing MHW and habitat‐complicity treatments with cages that exclude fish and large decapod predators. Finally, we also acknowledge that habitat complexity is a multi‐faceted driver of diversity, where the spatial scaling, arrangement of habitat‐elements, and the sizes of the habitat also affect epifaunal community structures (Tokeshi & Arakaki, 2012).

### 4.4 Rankings of test factors and interactions

Processes that control community dynamics are context dependent, making it a challenge to rank ecological test‐factors according to their relative importance and discover general ecological rules (Lawton, 1999). However, by using a four‐factorial experimental approach we could identify context dependency (i.e., significant statistical interactions) and rank, in order of importance and across animal responses, the test factors as season > intra‐season > habitat‐complexity > heating (by comparing sum of squares) (Underwood, 1990). Recently, Chen et al., (2021b) comprehensively reviewed the epifaunal literature but did not discuss interactions between and rankings of multiple ecological test factors. Still, some epifaunal studies have tested for ecological rules and context dependency, showing that community structures can be partially predicted by the density of primary and secondary foundation species and the environmental conditions associated with seasonality, intertidal elevation, subtidal depth, and latitude (Angelini et al., 2015; Bishop et al., 2012; Navarro‐Mayoral et al., 2020; Ravaglioli et al., 2021; Thomsen et al., 2016). For example, Navarro‐Mayoral et al., (2020) showed that epifauna were more diverse and abundant in summer and in shallow habitat compared to winter and deep habitats, likely because secondary foundation species were more common under the former conditions. Similarly, Thomsen et al., (2016) also showed positive density dependency of primary and secondary foundation species, with strongest effects observed in the transition zone between intertidal and subtidal habitats. In the present study we found several examples on context dependency, as facilitation was stronger in the warmer intra‐season during summer for both total epifaunal abundance and the copepods. Taken in concert, these results suggests that epifaunal responses may be predicted from relatively few fundamental environmental axes (Thomsen et al., 2022). More specifically, we aimed to test for interactions between MHWs and the spatiotemporal context. We only found significant interactions between MHWs vs. season and vs. habitat complexity for copepods (where more individuals inhabited heated plates in summer and when habitat complexity was high), implying that heatwave effects generally were consistent over experimental conditions. Still, other studies have found elevated heatwave effects when exposed to co‐occurring stressors, like high water turbidity (Tait et al., 2021), after coastal uplifts (Schiel et al., 2021), and when intertidal desiccation is also extreme (Thomsen et al., 2019). To date, observations and field experiments have largely focused on MHW effects under warmer summer conditions (Ashton et al., 2017; Clark et al., 2019; Smale et al., 2011, 2019), although globally, the frequency and intensity of winter and autumn heatwaves have also increased significantly (Thoral et al., 2022). It is therefore important to continue to test for interactions between heatwaves and seasons (and other stress‐factors) to better understand how marine communities may change in the future.

### 4.5 Study limitations

We tested if animals living around foundation species were affected by heatwaves using a single, species‐specific holdfast mimic for each of the seven seaweed species, thereby disregarding potential effects associated with intra‐specific variability and holdfast sizes, even though these attributes can affect epibiota communities (Hauser et al., 2006; Thomsen et al., 2018a; Tuya et al., 2011). Furthermore, the 3D printer resolution was likely coarser than natural holdfast structures, so the interstitial spaces in the mimics may be larger than in live holdfasts. However, using mimics allowed us to ensure test units had similar starting conditions, habitat age, material properties, colour, and that bacterial biofilm, epiphytes and epibiota were absent, to provide stronger unbiased tests of heatwave effects (Hauser et al., 2006; Myers & Southgate, 1980; Smith & Rule, 2002; Thomsen et al., 2022). Unlike live seaweed, abiotic mimics cannot be consumed by grazers, potentially limiting (and under‐representing) colonisation and survival by herbivores (Hauser et al., 2006; Hicks, 1986; Thomsen, Alestra, et al., 2018; Winkler et al., 2017). Our study was also limited spatially because we only did the experiments at a single ‘artificial’ location (a floating pontoon) and because mimics were positioned vertically on the pontoon, without being in direct contact with other reef habitats. This position implies that epifaunal colonisation probably occurred from swimming adults or larval dispersal, traits that vary considerably between taxonomic groups (Raimondi & Keough, 1990). For example, swimming copepods (and to a lesser extent amphipods) rapidly colonised our mimics, whereas gastropods were less common compared to gastropods found on reef‐associated seaweed, where they can crawl and disperse between neighbouring seaweed habitats (Edgar, 1992; Tanner, 2003; Thomsen et al., 2016, 2018a). Future work could also benefit from adopting trait‐based approaches that measures epifaunal traits, like individual sizes, growth rates, feeding guilds, or epifaunal mobility and dispersal rates, to supplement traditional taxonomic analyses, like done for invertebrates and fish in different coastal habitats (Henseler et al., 2019), estuarine infauna (Ellis et al., 2017), and seaweed‐associated epifauna (Stelling‐Wood et al., 2020). Finally, our study was also limited because we only tested a single, relatively short heatwave duration. Future studies should also quantify epifaunal colonisation and community dynamics using different magnitudes and durations of MHWs (particularly testing for longer MHWs), test for MHW effects on older epibiota communities (e.g., by incubating mimics for long periods prior to heating treatments), and test for lag‐effects (e.g. by measuring effects long after heating has ceased), as done in a few marine heatwave studies on sessile epifaunal communities, done over weeks (Smale et al., 2011), months (Clark et al., 2019), and even exceeding 1 year (Ashton et al., 2017).

## 5 CONCLUSION

Marine seaweed‐associated epifauna are essential components in marine food webs, but no studies have tested experimentally how MHWs affect these important communities under realistic field conditions. We therefore tested if epifauna were affected by experimentally induced MHWs, and if MHW impact was consistent within and between seasons, and with the complexity of co‐occurring foundation species. We found that taxonomic richness and total abundances of epifauna (dominated by copepods) were highest when animals were exposed to heatwaves, during summer and warmer intra‐seasonal conditions, and when secondary foundation species were morphologically complex. Future studies should test if similar results are found for live foundation species, after real MHWs and if results can be scaled to other biogeographical regions, in particular at polar and tropical latitudes.

## AUTHOR CONTRIBUTIONS

**Shinae Montie:** Conceptualization (equal); formal analysis (lead); investigation (lead); methodology (lead); visualization (equal); writing – original draft (lead); writing – review and editing (equal). **Mads S. Thomsen:** Conceptualization (equal); funding acquisition (lead); supervision (lead); visualization (equal); writing – review and editing (equal).

## CONFLICT OF INTEREST STATEMENT

The authors declare that the research was conducted in the absence of any commercial or financial relationships that could be construed as a potential conflict of interest.

## Supporting information


Table S1.

## Data Availability

The data that support the findings of this study are openly available in figshare at https://doi.org/10.6084/m9.figshare.24040521.v1.
