# Supplementary material for: Facilitation of animals is stronger during summer marine heatwaves and around morphologically complex foundation species
Source: Ecol Evol. 2023 Sep 17;13(9):e10512. doi: 10.1002/ece3.10512 (PMC10505761; doi:10.1002/ece3.10512)
Supplement: Supplementary file 1 — Table S1. [file ECE3-13-e10512-s001.docx]

**Supplement Material**

**Table S1.** Correlation analysis between holdfast size data (Table 1, i.e., length, width, surface area and planform area) against univariate invertebrate responses, using Spearman’s correlation coefficient and significance tests.

| **Row** | **Column** | **Correlation** | **P-value** |
| --- | --- | --- | --- |
| Surface Area (mm^2^) | Richness | 0.136 | 0.126 |
| Planform Area (mm^2^) | Richness | 0.112 | 0.208 |
| Length Max (mm) | Richness | -0.070 | 0.433 |
| Width Max (mm) | Richness | -0.113 | 0.205 |
| Surface Area (mm^2^) | Abundance | 0.245 | **0.005** |
| Planform Area (mm^2^) | Abundance | 0.222 | **0.012** |
| Length Max (mm) | Abundance | -0.121 | 0.175 |
| Width Max (mm) | Abundance | -0.177 | **0.045** |
| Surface Area (mm^2^) | Ab. Other | -0.215 | **0.015** |
| Planform Area (mm^2^) | Ab. Other | -0.262 | **0.003** |
| Length Max (mm) | Ab. Other | 0.234 | **0.008** |
| Width Max (mm) | Ab. Other | 0.228 | **0.010** |
| Surface Area (mm^2^) | Ab. Hexanauplia | 0.225 | **0.011** |
| Planform Area (mm^2^) | Ab. Hexanauplia | 0.189 | **0.033** |
| Length Max (mm) | Ab. Hexanauplia | -0.062 | 0.488 |
| Width Max (mm) | Ab. Hexanauplia | -0.168 | 0.058 |
| Surface Area (mm^2^) | Ab. Malacostraca | 0.147 | 0.098 |
| Planform Area (mm^2^) | Ab. Malacostraca | 0.161 | 0.070 |
| Length Max (mm) | Ab. Malacostraca | -0.085 | 0.341 |
| Width Max (mm) | Ab. Malacostraca | -0.110 | 0.214 |

**Figure S1.** Time series of sea surface temperature measurements at the edge of Lyttelton Harbour during the four experimental periods (summer warm intra-season, summer cold intra-season, winter warm intra-season, winter cold intra-season; highlighted in grey). Daily Optimally Interpolated Sea Surface Temperature (OISST) v2. 1 data downloaded from the National Oceanic and Atmospheric Administration (NOAA).

**
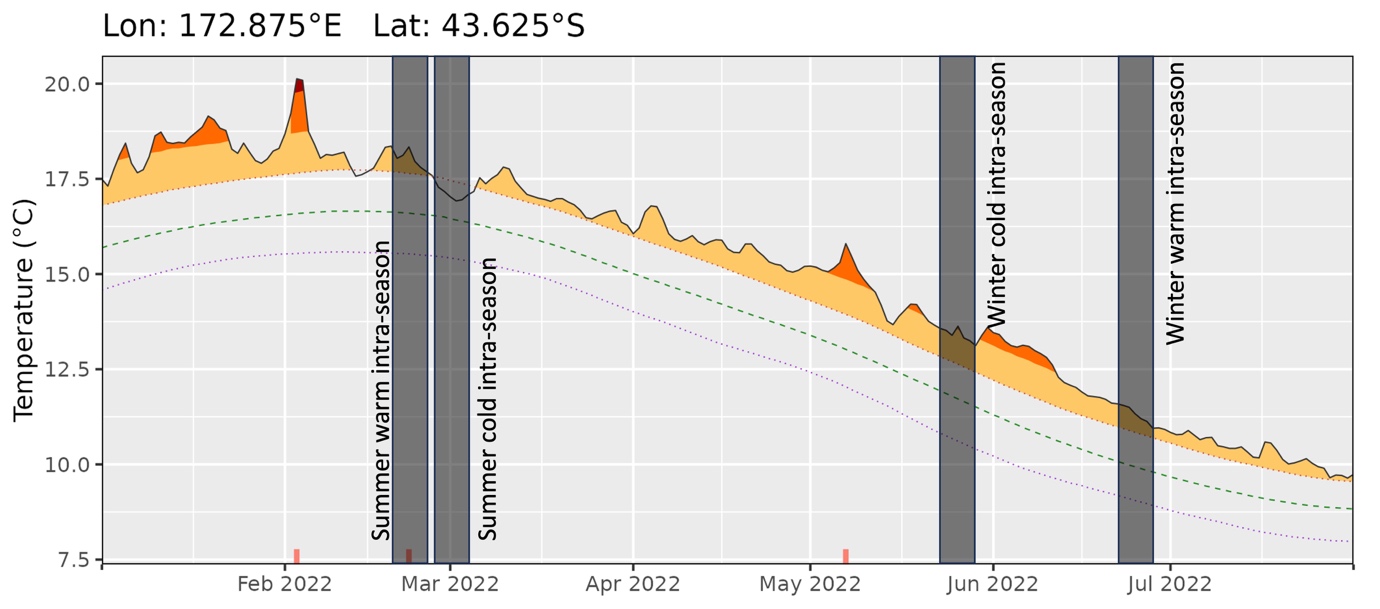
**

**Figure S2.** Correlation matrix between holdfast size data (Table 1, i.e., length, width, surface area and planform area) against univariate invertebrate responses, using Spearman’s correlation coefficient and significance tests. Correlation coefficient shown in black text accompanied by a red star for significant p-value (** = p < 0.01, * = p < 0.05).

**
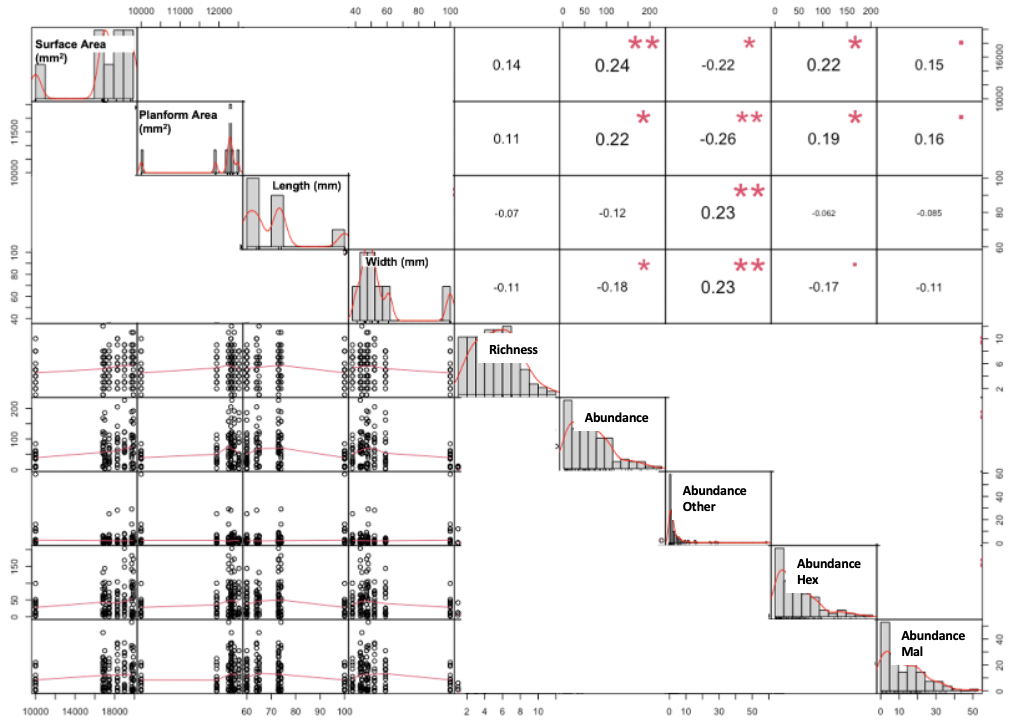
**

**Figure S3. Temporal changes in temperature under different habitat-complexities.** 1A. Average daily temperature (+/- SE) pooled across sub-experiments for control (blue) and heated (red) panels in summer and winter. Heated panels were on average 6.2°C and 5.1°C warmer than controls in the summer vs. winter, respectively. B. Average daily temperature (+/- SE) per species of holdfast mimic (*Carpophyllum maschalocarpum, Durvillaea antarctica, D. poha, D. willana, Ecklonia radiata, Macrocystis pyrifera* and *Undaria pinnatifida*) and control panels (no holdfast = turf only) pooled across sub-experiments, for control (blue) and heated (red) panels in summer and winter. Heated panels were on average 5.2-7.4°C and 4.6-5.6°C warmer than controls in summer vs. winter, respectively across all turf and turf + mimic combinations. The large discrepancy in average temperature for summer heated panels is due to a heat spike during the first two days of experiment one.
